# Developing iSWITCHED (Implementing SWITCHing EDucational Intervention): A Co-Designed Intervention to Support Safer Antipsychotic Switching in Severe Mental Illness

**DOI:** 10.1192/bjo.2025.10157

**Published:** 2025-06-20

**Authors:** Prachi Kaistha, Megan Beddow, Tom Kingstone, Ian Maidment, Saeed Farooq

**Affiliations:** 1 Keele University, Keele, United Kingdom; 2 Aston University, Birmingham, United Kingdom

## Abstract

**Aims:** Individuals with severe mental illness (SMI) face two–three times higher-risk of early death; over 60% are deaths linked to preventable physical health issues. Commonly prescribed antipsychotics (APs) like olanzapine, quetiapine, and risperidone effectively manage mental health (MH) symptoms but carry serious cardiometabolic risks. Although lower-risk APs have been available for nearly a decade, most patients remain on higher-risk APs as switching to improve cardio-metabolic side-effects is rarely implemented due to clinician uncertainty and relapse concerns. We aimed to co-design an educational intervention to support clinicians in evidence-based AP switching, incorporating input from clinicians, patients and caregivers.

**Methods:** Our intervention was co-designed iteratively, guided by Medical Research Council (MRC) framework for complex interventions. Work Package (WP) 1 formed exploratory basis for intervention development and included (i) two theory of change (ToC) workshops with clinicians (n=28) to identify barriers and opportunities for supporting people with SMI in switching APs; (ii) evidence review and synthesis of 32 clinical practice guidelines on switching APs; (iii) qualitative interviews with patients and caregivers (n=18) to explore perspectives on switching APs. Findings from WP1 were used to develop and refine intervention in WP2 iteratively through (i) two consensus-building workshops (CBWs) with clinicians and lay members (n=26); (ii) early user-feedback will be generated through ongoing think-aloud interviews and role-play activities.

**Results:** Insights from ToC workshops (28 MH clinicians), qualitative interviews (13 patients and five caregivers), and CBWs (22 MH clinicians and four lay members) highlighted importance of clear communication, collaborative clinician-patient relationships, clinician training, shared decision-making, and patient support while also addressing system-level barriers like poor integration and time constraints. The co-design approach established iSWITCHED, a five-component intervention to support clinicians in switching patients from higher-risk APs to lower-risk alternatives while promoting SDM between patients, carers, and clinicians. Intervention components include (i) a decision-aid that can be embedded in MH record systems to support clinicians in safely managing AP switches; (ii) peer-reviewed evidence-based guidelines for clinicians on AP switching; (iii) SDM guidelines to engage patients and carers; (iv) clinician training to enhance understanding and application of guidance and tool; (v) patient and clinician leaflets to support switching. Think-aloud interviews with 4 psychiatrists and pharmacists have been conducted so far to refine iSWITCHED.

**Conclusion:** The iSWITCHED switching intervention combines lived experiences, clinical expertise and integrates seamlessly into existing MH record systems. Before wider-implementation, it will be refined using insights from think-aloud interviews and role-play activities and piloted in a larger feasibility study.

